# The Dental Plaque Microbiome in Health and Disease

**DOI:** 10.1371/journal.pone.0058487

**Published:** 2013-03-08

**Authors:** Scott N. Peterson, Erik Snesrud, Jia Liu, Ana C. Ong, Mogens Kilian, Nicholas J. Schork, Walter Bretz

**Affiliations:** 1 J. Craig Venter Institute, Rockville, Maryland, United States of America; 2 Department of Biomedicine, Aarhus University, Aarhus, Denmark; 3 The Scripps Translational Science Institute and Scripps Health, La Jolla, California, United States of America; 4 Department of Cariology and Comprehensive Care, New York University, College of Dentistry, New York, New York, United States of America; Baylor College of Medicine, United States of America

## Abstract

Dental decay is one of the most prevalent chronic diseases worldwide. A variety of factors, including microbial, genetic, immunological, behavioral and environmental, interact to contribute to dental caries onset and development. Previous studies focused on the microbial basis for dental caries have identified species associated with both dental health and disease. The purpose of the current study was to improve our knowledge of the microbial species involved in dental caries and health by performing a comprehensive 16S rDNA profiling of the dental plaque microbiome of both caries-free and caries-active subjects. Analysis of over 50,000 nearly full-length 16S rDNA clones allowed the identification of 1,372 operational taxonomic units (OTUs) in the dental plaque microbiome. Approximately half of the OTUs were common to both caries-free and caries-active microbiomes and present at similar abundance. The majority of differences in OTU’s reflected very low abundance phylotypes. This survey allowed us to define the population structure of the dental plaque microbiome and to identify the microbial signatures associated with dental health and disease. The deep profiling of dental plaque allowed the identification of 87 phylotypes that are over-represented in either caries-free or caries-active subjects. Among these signatures, those associated with dental health outnumbered those associated with dental caries by nearly two-fold. A comparison of this data to other published studies indicate significant heterogeneity in study outcomes and suggest that novel approaches may be required to further define the signatures of dental caries onset and progression.

## Introduction

Dental caries is the single most common disease in childhood with a prevalence rate five times higher than the next most prevalent disease, asthma [1]. Dental caries remains a significant public health issue in children worldwide. Over 50% of children in the United States 5-to 9-years-old have at least one cavity or filling, and that increases to 78% among 17-year-olds [2]. These adverse outcomes are disproportionately present in disadvantaged segments of the United States population including children, adults and the elderly. It has been shown that dental care is the most common unmet need among children in the United States [3], [4].

Microbiological strategies to identify associations related to dental caries are limited due to the lack of highly selective media formulations and the uncultivability of many oral species. These approaches have implicated mutans streptococci and *Lactobacillus* spp. as signatures of childhood dental caries [5]–[10]. The more recent use of culture-free methods has alleviated many of these barriers. Surveys of microbial diversity in the human oral microbiota based on culture-free, clonal analysis of 16S rDNA gene sequence and phylogeny have estimated that greater than 700 microbial species inhabit the oral cavity [11]–[16]. The amount of 16S rDNA profiling data pertaining to the dental caries disease model is limited but growing rapidly [5], [10], [17]–[19]. Becker and colleagues [18] and Aas et al., [17] used 16S rDNA analysis of caries-free and caries-active children and adults to reveal species associated with dental health such as *Streptococcus sanguinis* and genera associated with dental caries in both primary and permanent dentitions such as *Streptococcus mutans* and low-pH, non-mutans streptococci, *Veillonella* spp., *Actinomyces* spp., *Bifidobacterium* spp., *Lactobacillus* spp., *Propionibacterium* spp., and *Atopobium* spp. Munson et al., [10] and Chhour et al., [5] reported that the predominant microbes in a small number of adults with advancing, deep caries lesions were *S. mutans* and *Lactobacillus* spp. but also included the genera *Prevotella*, *Selenomonas*, *Dialister*, *Fusobacterium*, *Bifidobacterium*, and *Pseudoramibacter* [5], [10]. Corby and colleagues employed 16S rDNA profiling of dental caries-associated flora in infants and children who were caries-active (n = 86) and caries-free (n = 118) [19]. *S. mutans*, *Actinomyces* spp., and *Lactobacillus* spp. were over-abundant in caries-active subjects whereas beneficial species associated with dental health included *Streptococcus parasanguinis, Abiotrophia defectiva, Streptococcus mitis, Streptococcus oralis*, and *S. sanguinis*. Taken together, the number of species/OTUs identified in these studies ranged from 75 to 197.

More recent studies have surveyed various domains in the oral environment in greater depth [15], [20], [21]. These surveys have shed light on the high-level of species diversity present in oral bacterial communities associated with both health and disease. A study focused on establishing associations of severe early childhood caries (ECC) using the 454 sequencing platform identified taxa associated with caries including: *Granulicatella elegans, Veilonella atypica, Veilonella* spp. HOT-780 [22]. Using species-specific PCR primers, *S. mutans and Bifidobacteriaceae* spp. were both identified as caries-associated. Species associated with dental health included: *Capnocytophaga gingivalis, Abiotrophia defectiva, Lachnospiraceae* spp. *Streptococcus cristatus* and *S. sanguinis.* Among the 3,802 high quality sequences analyzed (1,846 ECC, 1,956 caries-free), less than 50% of the observed OTU’s were common between ECC (n = 39) and healthy subjects (n = 41). Another study of severe childhood caries in permanent teeth examined caries progression to establish relationships between the bacterial communities present and the severity of caries [23]. Potential acid producers such as *Selenomonas* spp., *Neisseria* and *S. mitis* were more prevalent in the caries group whereas *Propionibacterium* FMA5 was significantly associated with caries progression but was not observed at high levels in these subjects. An overall reduction in species diversity in communities was observed as caries progressed, including the reduced abundance of *S. mitis* group, *Corybacterium matruchotii, Streptococcus gordonii, Streptococcus cristatus, Capnocytophaga gingivalis, Eubacterium* IR009, *Campylobacter rectus* and *Lachnospiraceae* sp. C1.

One of the central challenges associated with the characterization of human microbiota is the ability to cope with the enormous dynamic range of individual species and genome abundance. Random sampling strategies reveal only the surface layer of the true underlying complexity. All previously published studies analyzing full-length 16S rDNA have been limited due to shallow depth of clonal analysis. We elected to conduct a deep survey using Sanger sequencing of nearly full-length 16S rDNAs in order to evaluate and compare OTUs at the species level. The purpose of this study was to establish a comprehensive profiling of dental plaque to better understand the distinctions between microbial biofilms derived from caries-free (C-F) and caries-active (C-A) dental plaque. We employed a normalization strategy to enable the observation of less abundant members of the dental plaque community. Our study examined dental plaque derived from children 5–7 years of age from a single geographical region. It remains to be seen to what extent the dental plaque microbiome defined here differs from that of the human population as a whole. Our study represents an appropriate framework for comparative purposes to address these questions. It is anticipated that such efforts will eventually allow us to determine whether a core microbiome may be defined that describes the microbial community common to all human subjects. As culture-independent sequencing studies continue to accumulate, it appears that each identifies a number of unique associations that differ from those described previously, suggesting that caries onset and progression is complex. It is conceivable that the microbial signatures of dental health and disease are not the same around the world. The species contributing to caries health and disease may be largely interchangeable and therefore numerous species signatures may ultimately be needed to define a C-F and C-A dental plaque microbiome. There is still much to learn concerning how these communities contribute to pH homeostasis, maintenance of health, disease onset and progression.

## Materials and Methods

### Dental Caries Phenotype Determination

Dental caries examinations were performed on 8 subjects (5 female, 3 male) that were C-A (n = 4) and C-F (n = 4) respectively. These subjects (5–7 years old) were medically healthy and presented with only primary dentition. This group of children resides in the suburbs of the city of Montes Claros, State of Minas Gerais, Brazil. Water fluoride levels in this city are less than optimal (<0.7 ppm) and dental check-ups for this group were negligible.

### Ethics Statement

Parents signed informed consent approved by New York University and UNIMONTES (State University of Montes Claros) institutional review boards after the children assented.

### Dental Caries Examinations

**Figure 1:**
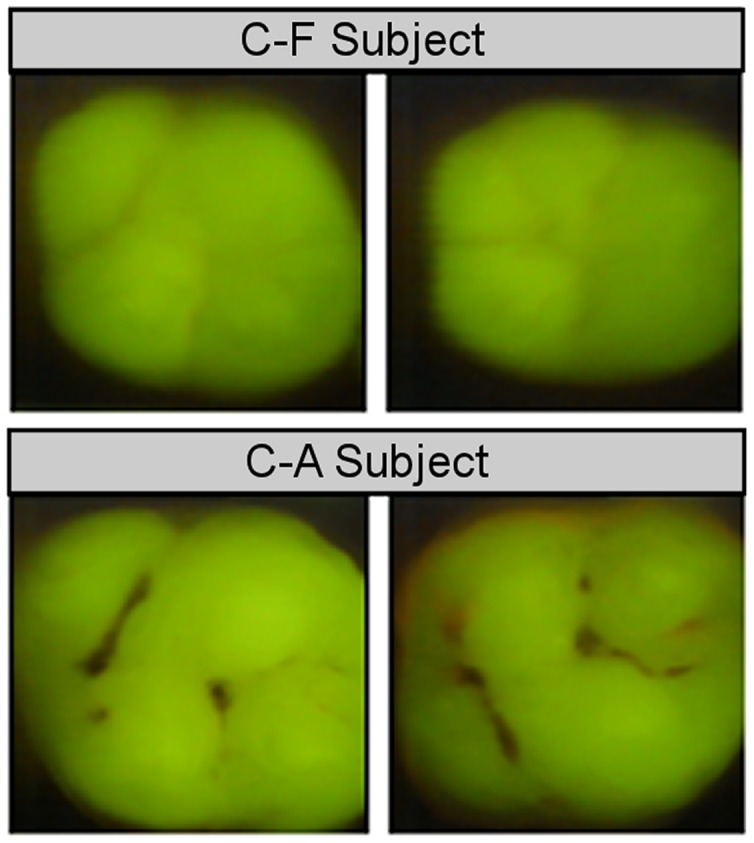
Caries Phenotype Determination. Quantitative Light fluorescence (QLF) of occlusal surfaces of C-F and C-A subjects. Quantitative light fluorescence (QLF) examination was conducted to complement clinical examinations and the DIFOTI procedure. C-A subjects had an average of 12 (range = 6–17) decayed tooth surfaces whereas C-F subjects presented with a decay component = 0. Subjects had an average of 11.5 decayed surfaces and 2.3 restored surfaces. These individuals presented with primary dentition only. C-A subjects had an average of 74 healthy surfaces.

We used a combination of three dental caries exams for accurate characterization of dental caries phenotypes in C-F and C-A subjects. These included: 1) Clinical examination of dental caries in all teeth, assessed with the aid of artificial light and a dental mirror according to NIDCR criteria [24] to include white spot lesions and cavitated lesions; 2) Digital imaging fiber-optic trans-illumination (DIFOTI) recorded images of dental lesions (incipient and frank lesions) to complement the caries clinical examination [25]; 3) Quantitative light fluorescence (QLF) profiled images of dental lesions [26] similar to the DIFOTI procedure that are not readily captured by visual examinations and complemented the caries clinical examination. C-A subjects had an average of 12 (range = 6–17) decayed tooth surfaces whereas C-F subjects presented with a decay component = 0 (**Figure 1**).

### Dental Plaque Biofilm Sampling

Subjects were instructed to refrain from brushing or eating prior to sampling. Dental plaque samples were obtained using a sterile toothbrush passed slowly across all tooth surfaces in order to maximize sample collection. We elected to collect an overall plaque sample of the entire dentition rather than sampling site-specific surfaces that are associated with health or disease to enable characterizations that would otherwise be biomass limited. Moreover, our previous studies demonstrate that the dental microbiota associated with localized healthy tooth surfaces and caries lesions are similar within the same oral cavity [19]. Dental plaque was dislodged from the toothbrush by agitation for 1 minute into tubes containing 8 mL of sterile reduced transport fluid (RTF) [27] held at 4°C prior to storage at −80°C.

### Genomic DNA Isolation from Dental Plaque

Dental plaque samples were thawed and 1 mL of the cell mixture was used for DNA isolation as described by Turnbaugh et al., [28]. In order to achieve uniform lysis of cells we used mechanical disruption with a bead beater (BioSpec Products, Bartlesville, OK) in the presence of 2% SDS, phenol:chloroform and 0.1-mm-diameter zirconia/silica beads (BioSpec Products). The lysate was extracted one additional time and genomic DNAs were precipitated with isopropanol. The genomic DNAs were further purified using the MasterPure DNA Purification Kit (Epicentre Biotechnologies, Madison, WI). The genomic DNA was evaluated subjectively on agarose gels and quantitated using a UV spectrophotometer. The average yield of genomic DNA from plaque samples was ∼100 µg/subject. The yields of DNA from each dental plaque were non-limiting and similar in C-F and C-A subjects.

### Re-association Kinetics Enrichment (RAKE) of Moderate and Low Abundance Genomic DNAs

We combined genomic DNAs (10 µg each) isolated from 4 C-A and 4 C-F subjects to create two reference pools (C-A and C-F). The C-A and C-F genomic DNA pools in re-association buffer (50 mM HEPES, pH 7.5, 200 µM EDTA) were sheared to 3–8 Kb by nebulization. The samples were heat denatured in a volume of 10 µl at 95°C for 5 minutes, NaCl was added to 0.5 M, and then held at 65°C. Hydroxyapatite (HA) columns were prepared using ∼20 cm^3^ of resin (BioRad, Irvine CA) and were maintained at 65°C using a water jacket for optimal binding and nucleic acid migration. The columns were equilibrated sequentially with 10 mL of sodium phosphate buffer (SPB); Wash 1 (480 mM SPB), followed by wash 2 (120 mM SPB) and wash 3 (30 mM SPB) all at 65°C. At various timepoints (0–60 hours), the DNAs were diluted 100-fold in 30 mM SPB and loaded onto hydroxyapatite columns to separate and recover ssDNA and dsDNA. The ssDNA was eluted using 120 mM SPB followed by elution of dsDNA with 480 mM SPB. The DNAs were then precipitated, washed, resuspended in TE, and quantitated using a Nanodrop spectrophotometer (Thermo Scientific, Wilmington, DE). This procedure resulted in four distinct genomic DNA fractions from both C-F and C-A genomic DNA pools: **1.** non-enriched, **2.** 64-fold enriched (4 hours re-association), **3.** 1,000-fold enriched (24 hours re-association) and **4.** 10,000-fold enriched (60 hours re-association). These determinations were made by performing qPCR and comparing the number of cycles required for ssDNA and dsDNA fractions to cross the fluorescence threshold.

### 16S rDNA Amplicon and DNA Sequence Generation

The recovered ssDNA fractions were used as a template in PCR reactions using the universal primers 8F/1492R to generate nearly full-length 16S rDNA amplicons. The 16S rDNA amplicons were cloned into a TOPO cloning vector, pZErO (Invitrogen Inc, Carlsbad, CA) to generate 8 libraries for random shotgun sequencing as follows: non-enriched C-F and C-A, 64-fold enriched C-F and C-A, 1,000-fold enriched C-F and C-A, and 10,000-fold enriched C-F and C-A. All clones were sequenced using the Sanger method run on an ABI 3730 XL sequencing instrument. The average read length of clones (paired-end reads) was 992 bases. Eight libraries, two of which were sequenced in two independent sequence runs were submitted to the trace archive division at GenBank with the following accessions: C-F unenriched: 2334480902–2334499005, C-A unenriched: 2334532826–2334550317 269128 C-F unenriched-2∶2334458758–2334468399, C-A unenriched-2∶2334499006–2334512867, CF 64-fold enriched: 2331272350–2331277318, CA 64-fold enriched: 2331279180–2331284211, CF 1000-fold enriched: 2334468400–2334480901, CA 1000-fold enriched: 2334512868–2334521646, CF 10,000-fold enriched: 2331270338–2331272349, CA 10,000-fold enriched: 2331277319–2331279179.

### 16S rDNA Phylogenetic Analysis and OTU Determination

The 16S rDNA paired-end sequences were processed using the JCVI internal 16S rDNA data analysis pipeline. Briefly, short reads, vector sequences, and sequences of poor quality were filtered. Sequences were then screened for possible chimeras using Chimera Slayer [29] and were removed from further analysis. We next clustered the sequences using the cd-hit-est based clustering method [30]. Operational taxonomic units (OTUs) were defined using a 97% sequence similarity cutoff, roughly corresponding to species-level groupings. Sequences were grouped into distinct OTUs using Felsenstein-corrected similarity matrices such that the least similar pair within an OTU shared at least 97% similarity across its length. The Ribosomal Database Project (RDP) Classifier [31] was used to classify the 16S rDNA sequences into the the most detailed taxonomic category by aligning 16S rDNA sequences to a curated database of taxonomically annotated sequences at the resolution of genus-level assignments. In order to achieve taxonomic assignments to the species level, all of the 16S rDNA sequences were mapped to the curated 16S rDNA database (RDP v10.0) using BLASTN. Sequences with less than 97% similarity to sequences in the RDP database were considered novel reads, whereas those with greater than 97% identity were used to associate a group of OTUs to specific species. An OTU is considered to be a novel OTU only if all members belonging to that OTU failed to be assigned to a known OTU.

### Statistical Analysis

The relative abundance of each OTU was computed by dividing the counts of individual OTUs associated with each pool by the total number of OTUs identified from the 16S rDNA profiling. We thus established a relative species frequency for the C-F and C-A pools. A standard z-test of the equality of frequencies was used to determine the significance of the difference in relative OTU frequencies between the C-A and C-F pools. Given that multiple OTUs were assessed for frequency differences, we accommodated the resulting multiple comparisons by computing false discovery rates [32], [33] (http://cran.r-project.org/web/packages/qvalue/).

## Results

We performed a deep survey of the microbial composition of the dental plaque microbiome derived from children (ages 5–7) that were either C-F or C-A as determined by clinical examination, DIFOTI and QLF for accurate phenotype characterization to establish its population structure and to enable a comprehensive comparison of the species/OTUs present in C-F and C-A subjects (**Figure 1**). In total we sequenced 53,038 16S rDNAs derived from C-F and C-A pooled samples yielding 47,198 clone sequences (C-F = 21,705 and C-A = 25,493) (**Table 1**). The majority of clones analyzed (66%) were from unnormalized libraries, whereas additional sequencing was performed on clones derived from normalized libraries using a procedure we refer to as Re-Association Kinetic Enrichment (RAKE), a normalization procedure that is based on C_o_t analysis, wherein the re-association rate of denatured double stranded DNAs is dependent on the concentration of DNA sequences in the mixture (see Materials and Methods). This allowed us to perform a more comprehensive analysis of dental plaque microbiota.

**Table 1:** 16S rDNA Libraries for C-F and C-A Pools.

Library	C-F	C-A	Total
non-enriched	13,530	18,295	31,825
64-fold enriched	1,618	1,667	3,285
1,000-fold enriched	6,052	4,991	11,043
10,000-fold enriched	505	540	1,045
**Total**	**21,705**	**25493**	**47,198**

The combined (C-F and C-A) results of the 16S rDNA analysis indicate several interesting points regarding the diversity and community structure of the dental plaque microbiota. In total, we identified 1,372 OTUs, 984 OTUs in the C-F microbiome and 1,014 OTUs in the C-A microbiome. This is by far the largest number of OTUs identified in the dental plaque microbiome using full-length 16S rDNA sequence data. The data indicate that the species present in the dental plaque microbiome span at least four orders of magnitude in abundance. A total of 626 OTUs were common to both microbiomes. A total of 375 OTUs were observed in ten or more clone sequences. The OTUs derived exclusively from C-F (358 OTUs) or C-A (388 OTUs) microbiomes were generated from 790 and 750 16S rDNA clones respectively, indicating that these OTUs correspond to very low abundance phylotypes. This observation indicates that the primary difference between C-F and C-A subjects is not related to the “appearance” of unique phylotypes, but rather the altered abundance of particular phylotypes.

### Taxonomic Characterization of the Dental Plaque Community

The microbial diversity present in dental plaque is not randomly distributed among bacterial taxa. Among the ∼70 previously defined phyla, only ten were observed in our survey. Moreover only four of these phyla are present in abundance greater than 1% of the total. The dominance of the Firmicutes is striking and comprises more than 82% of the surveyed community. Nearly 66% of the Firmicutes represent *Streptococcus* spp., corresponding to a large number of named and uncharacterized phylotypes. This observation reflects the massive expansion of this genus in dental plaque biofilms and emphasizes its overall importance with respect to the biochemical flux and maintenance of homeostasis of the biofilm community. The next most abundant phylum is Proteobacteria (11%) followed by Bacteriodetes (3%) and Fusobacteria (2%). Additional phyla observed at less than 1% included: Actinobacteria, Cyanobacteria, Deferribacteres, Spirochaetes, SR1 and TM7. Despite the large number of OTUs identified, these belonged to a limited number of only 36 genera (5 or more clones, ∼0.01%). Only 10 genera were observed at high abundance (>1%). These included: *Streptococcus* (52%), *Veillonella* (8.5%), *Granulicatella* (7.2%), *Fusobacterium* (5.6%), *Neisseria* (5.5%), *Campylobacter* (4.7%), *Gemella* (3.7%), *Abiotrophia* (2.6%), *Selenomonas* (1.5%) and finally *Capnocytophaga* (1%). It is potentially interesting to note that excepting the *Streptococcus,* the most abundant genera display a narrow range of relative abundance (2.1–4%).

### Population Structure of the Dental Plaque Microbiota

The 1,372 OTUs observed corresponded to 205 named species and 44 genus level assignments (**Figure S1**). We observed two dominant species present in the dental plaque samples profiled here, *S. mitis* (25.5%) and *S. sanguinis*, (9.1%). There were 14 additional species present at abundance levels greater than 1% in the combined data. These included: *Veillonella parvula* (7.5%), *Streptococcus oralis (*6.1%), *Neiserria subflava* (3.0%), *Gemella haemolysans* (2.8%), *Granulicatella elegans* (2.6%), *Streptococcus gordonii* (2.6%), *Abiotrophia defectiva* (2.5%), *S. cristatus* (1.9%), *C. gracilis* (1.8%), *C. showae* (1.4%), *S. infantis* (1.3%), *S. constellatus* (1.3%), *S. mutans* (1.2%) and *C. concisus* (1.1%). This composition is consistent with data generated as part of the HMP consortium surveys [34]–[36]. Collectively, these 16 species comprise approximately 71% of the bacterial biomass of the dental plaque biofilms surveyed here. The biochemical flux occurring in dental biofilms is strongly influenced by the metabolic activities of these species. We examined an additional 129 OTUs representing moderately abundant OTUs (0.1–1%) and found that it reflected a similarly high representation of *Streptococcus* spp. and included a disproportionately large number of Proteobacteria, namely *Neisseria* spp. and *Campylobacter* spp. We observed ∼400 OTUs that we classify as low abundance phylotypes (0.01–0.1%). The remaining ∼800 OTUs observed represent very low abundance phylotypes, present at <0.01% of the total microbiome. The depth of our sequencing efforts limit the extent that we can project the true size and diversity of the very low abundance species constituting the long tail of the population structure. The population structure of the dental plaque microbiome therefore appears to represent a very small group of dominant species that display only modest phylogenetic diversity (seven genera) followed by approximately one hundred moderately abundant species with increased phylogenetic diversity. Finally, the less abundant phylotypes define the highest level of phylogenetic diversity in the population.

### Differential Abundance of Genera Associated With C-F and C-A Microbiota

**Figure 2:**
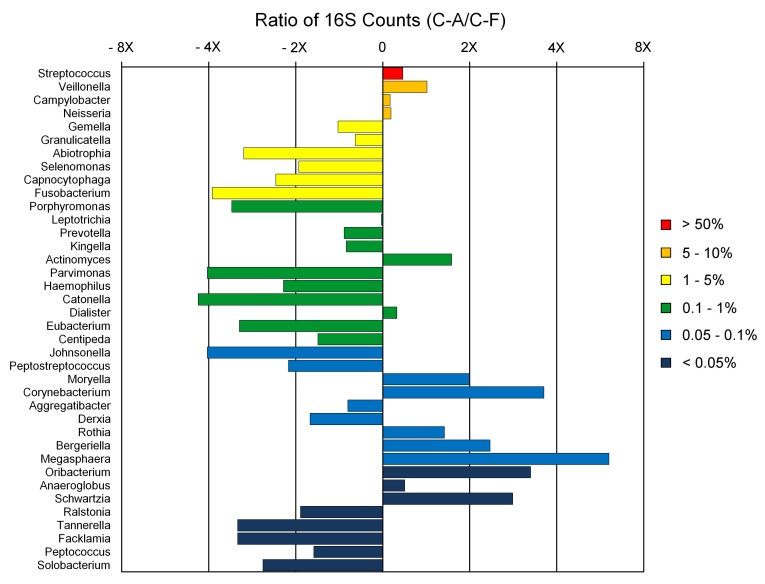
Differential Representation of Genera in the C-F and C-A Microbiomes. Bars to the left correspond to over-representation of genera in the C-F subject pool, and bars to the right correspond to over-representation in the C-A subject pool. Bar color key references percentage of the total 16S sequences.

We examined the similarities and differences of genera present in C-F and C-A subject pools as depicted in **Figure 2** **.** Nearly two-thirds of the observed genera are over-represented in the C-F microbiome, however the four most abundant genera including *Streptococcus* spp., *Veillonella* spp., *Campylobacter* spp. and *Neisseria* spp. are all over-represented in the C-A subject pool. The observed differential representation of these genera is small in each case but potentially meaningful given the high abundance of these genera in the community. Among the moderately abundant genera, virtually all are over-represented in the C-F microbiota except for the *Actinomyces* spp. and *Eubacterium* spp. The last group of genera, representing those of low abundance display greater variability when comparing C-F and C-A microbiota.

### Taxonomic Analysis of Differentially Abundant Phylotypes in C-F and C-A Microbiota

**Figure 3:**
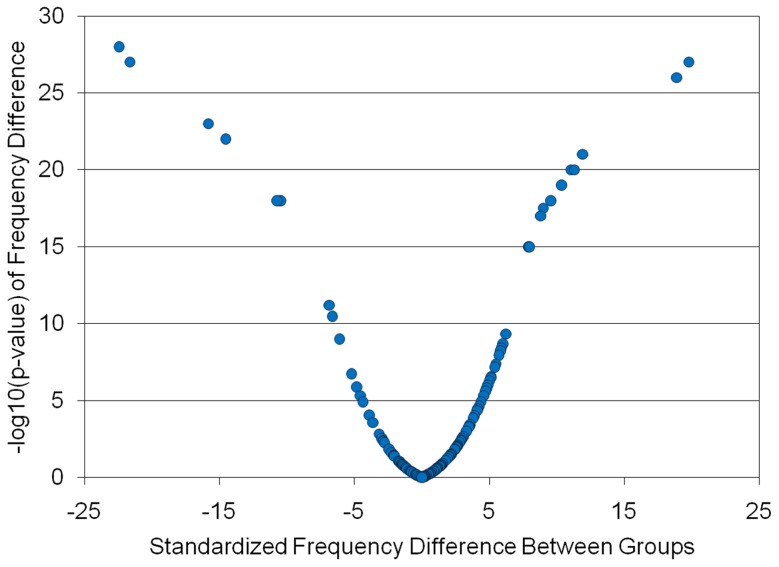
Statistical Significance of Differential Species Abundance Between the C-F and C-A Microbiomes. Volcano plot depicting the relationship between –log p-values resulting from standard binomial tests of frequency differences between C-A and C-F for each phylotype identified as a function of the standardized difference in phylotype frequency between C-A and C-F.

We compared the species profile of the C-F and C-A subject pools to identify differentially abundant phylotypes. The most compelling observation when comparing the OTUs derived from C-F and C-A dental plaque is the remarkable uniformity in abundance of the vast majority of represented phylotypes. This uniformity is likely to reflect a strong selective pressure that has been applied over millions of years of co-evolution that acts to maintain the species homeostasis of the dental plaque microbiome. We used standard z-tests of equality of phylotype frequencies between C-A and C-F subject pools to identify those exhibiting the greatest differences. False discovery rate calculations suggested that 87 phylotypes were strongly associated with either caries health or disease and these species had less than a 1% chance of being a false positive result despite the multiple comparisons (p = <0.05). **Figure 3** provides a Volcano plot depicting the probabilistic significance of the frequency differences (-log p-values from the z-tests) as a function of the standardized difference in phylotype abundances. It can be seen that for some phylotypes, the abundance differences are very large and have a very low probability of having occurred by chance.

**Figure 4:**
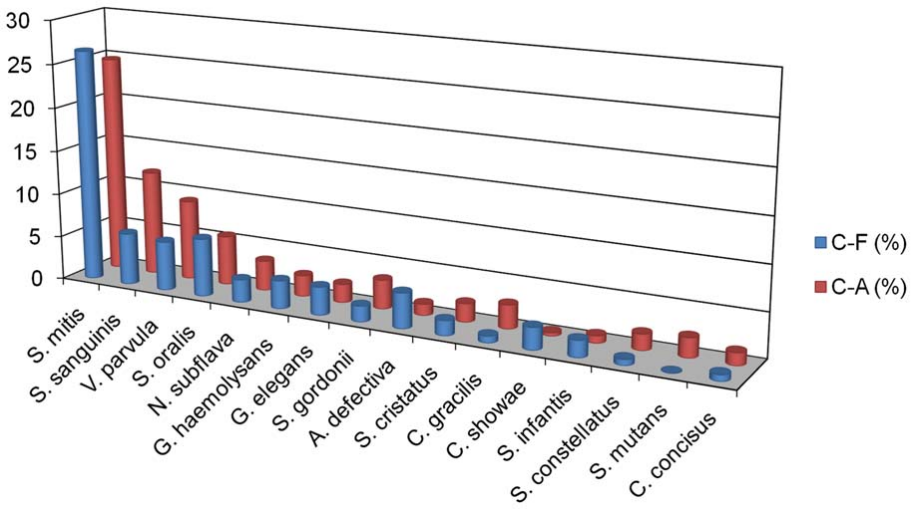
Comparison of C-F and C-A Species Abundance. The 16 most abundant species and their relative abundance in C-F and C-A subject pools.

We analyzed the relative abundance of phylotypes present in C-F and C-A subject pools. In total we identified 87 differentially represented phylotypes comparing C-F and C-A 16S rDNA profiles (**Table 2** **and Table S1**). It is of note that among the most abundant species in dental plaque biofilms, all (16 phylotypes >1% abundance) were differentially abundant in C-F compared to C-A microbiomes (**Figure 4**). Moreover, 11 of the 16 species were over-represented in the C-A subject pool. This observation is in contrast to the moderately abundant phylotypes (0.1–1%) where the number of species over-represented in the C-F subject pool is 26 compared to 10 phylotypes over-represented in the C-A subject pool and 14 phylotypes that displayed no significant difference in abundance.

**Table 2:** Phylotypes Differentially Represented in C-F and C-A Dental Biofilms.

Total	C-F	C-A	Phylotype	p-value	FDR
47198	21705	25493	Bacteria		
4325	1288	3037	*Streptococcus sanguinis*	0	0
3520	1205	2315	*Veillonella parvula*	0	0
1220	697	523	*Granulicatella elegans*	0	0
1220	381	839	*Streptococcus gordonii*	0	0
1189	867	322	*Abiotrophia defectiva*	0	0
865	168	697	*Campylobacter gracilis*	0	0
643	544	99	*Campylobacter showae*	0	0
633	420	213	*Streptococcus infantis*	0	0
601	146	455	*Streptococcus constellatus*	0	0
578	8	570	*Streptococcus mutans*	0	0
359	269	90	*Fusobacterium nucleatum*	0	0
350	266	84	*Selenomonas infelix*	0	0
250	190	60	*Streptococcus australis*	0	0
141	118	23	*Catonella morbi*	0	0
93	85	8	*Veillonella criceti*	0	0
81	73	8	*Capnocytophaga leadbetteri*	0	0
511	158	353	*Campylobacter concisus*	6.358E−12	2.500E−11
225	54	171	*Kingella denitrificans*	3.282E−11	1.220E−10
76	62	14	*Veillonella sp.*	4.642E−10	1.640E−09
48	1	47	*Streptococcus sobrinus*	1.020E−09	3.430E−09
62	52	10	*Porphyromonas sp.*	2.109E−09	6.780E−09
88	68	20	*Capnocytophaga sputigena*	3.765E−09	1.160E−08
1302	702	600	*Gemella haemolysans*	5.810E−09	1.710E−08
96	72	24	*Neisseria elongata*	1.133E−08	3.200E−08
48	41	7	*Abiotrophia sp.*	4.158E−08	1.130E−07
77	59	18	*Parvimonas micra*	6.722E−08	1.760E−07
1421	557	864	*Neisseria subflava*	1.845E−07	4.660E−07
26	25	1	*Prevotella sp.*	2.837E−07	6.920E−07
101	72	29	*Lautropia mirabilis*	3.270E−07	7.710E−07
49	40	9	*Fusobacterium canifelinum*	5.468E−07	1.250E−06
74	55	19	*Neisseria sp.*	9.834E−07	2.170E−06
370	124	246	*Streptococcus salivarius*	1.344E−06	2.880E−06
47	38	9	*Selenomonas dianae*	1.601E−06	3.330E−06
72	53	19	*Gemella sanguinis*	2.517E−06	5.090E−06
273	88	185	*Streptococcus intermedius*	4.817E−06	9.410E−06
58	44	14	*Neisseria bacilliformis*	4.925E−06	9.410E−06
12047	5747	6300	*Streptococcus mitis*	1.168E−05	2.170E−05
76	16	60	*Leptotrichia wadei*	1.271E−05	2.250E−05
31	26	5	*Campylobacter rectus*	2.300E−05	3.970E−05
2864	1424	1440	*Streptococcus oralis*	3.526E−05	5.940E−05
112	73	39	*Porphyromonas catoniae*	4.503E−05	7.400E−05
18	0	18	*Corynebacterium matruchotii*	9.021E−05	1.450E−04
71	49	22	*Leptotrichia sp.*	9.780E−05	1.540E−04

#### Actinobacteria

Four members of this taxon were differentially abundant in our survey. *Propionibacterium propionicum* was exclusively observed in the C-F subject pool, whereas *Corynebacterium matruchotii* was exclusively observed in the C-A subject pool. Two *Actinomyces, A. naeslundii* and an uncharacterized species were both over-represented in the C-A subject pool.

#### Bacteroidetes

Among the twelve members of this phylum that were differentially represented, all but two were over-represented in the C-F subjects pool, namely *Prevotella oulorum* and *Prevotella salviae.* The remaining members of this group include: two *Porphyromonas* spp.: one uncharacterized species and *P. catoniae*, seven *Prevotella* spp. including *P. tannerae*, one uncharacterized phylotype, *P. nanceiensis, P. saccharolytica* and *P. intermedia* and finally three *Capnocytophaga* spp. *C. leadbetteri, C. sputigena* and *C. granulosa.*

#### Firmicutes: Gemella, Abiotrophia, Granulicatella

Four *Gemella* phylotypes were differentially abundant and all were over-represented in the C-F subject pool, including: *G. haemolysans.*, *G. sanguinis*, *G. morbillorum* and *G. bergeri*. Two *Abiotrophia* spp. were both over-represented in the C-F subject pool including *A. defectiva* and an uncharacterized *Abiotrophia* spp. Two *Granulicatella* spp. were differentially abundant in our survey, *G. elegans* was over-represented in the C-F subject pool, whereas *G. adjacens* was over-represented in the C-A subject pool.

#### Firmicutes: Veillonella, Eubacterium, Parvimonas, Mogibacterium, Catonella, Peptostreptococcus, Centipeda, Dialister, Megasphaeara, Mitsuokella, Selenomonas, Solobacterium

Among the four differentially abundant *Veillonella* spp. observed, two were over-represented in the C-F subject pool including: *V. criceti* and an uncharacterized phylotype. The remaining two phylotypes, *V. parvula and an* uncharacterized phylotype were over-represented in the C-A subject pool. Among the four differentially abundant *Eubacterium* spp. three were over-represented in the C-F subject pool including: *E. brachy, E. yurii* and an uncharacterized *Eubacterium* spp. *E. saburreum* was over-represented in the C-A subject pool. *Parvimonas micra, Mogibacterium timidum, Catonella morbi, Peptostreptococcus stomatis, Centipeda periodontii, Dialister pneumosintes* and *Solobacterium moorei* were all over-represented in the C-F subject pool. *Megasphaera micronucliformis* and an uncharacterized *Mitsuokella* spp. were over-represented in the C-A subject pool. Finally, three *Selenomonas* spp. were differentially abundant, *S. infelix* and *S. dianae* were over-represented in the C-F subject pool, and an uncharacterized *Selenomonas* spp. was over-represented in the C-A subject pool.

#### Firmicutes: Streptococcus

Among the 18 differentially abundant phylotypes in this genus only four were over-represented in the C-F subject pool including: *S. infantis*, *S. australis*, *S. oligofermentans,* and *S. suis.* The remaining 14 phylotypes were over-represented in the C-A subject pool including: *S. sanguinis*, *S. mutans*, *S. sobrinus*, *S. mitis*, *S. intermedius*, *S. gordonii*, *S. parasanguinis*, *S. constellatus*, *S. cristatus*, *S. oralis*, *S. equi, S. dentirousetti* and *S. peroris*. *S. mutans* displayed the largest differential abundance compared to any other phylotype observed.

#### Fusobacteria: Fusobacterium and leptotrichia

All three differentially represented *Fusobacterium* spp. were over-represented in the C-F subject pool: *F. periodonticum, F. canifelinum* and *F. nucleatum*. Three *Leptotrichia* spp. were differentially represented in our survey, two were over-represented in the C-F subject pool including: *L. shahii* and an uncharacterized *Leptotrichia* spp. *L. wadei* was over-represented in the C-A subject pool.

#### Proteobacteria: Kingella, Neisseria Simonsiella, Campylobacter, Cardiobacter, Haemophilus and Lautropia

*Kingella denitrificans* was over-represented in the C-A subject pool. Six *Neiserria* spp. were differentially abundant in our survey. Five of these were over-represented in the C-F subject pool including: two uncharacterized *Neiserria* spp., *N. elongata, N. bacilliformis* and *N. lactamica. N. subflava* was over-represented in the C-A subject pool. *Simonsiella muelleri* was over-represented in the C-F subject pool. Among the six differentially abundant *Campylobacter* spp. four were over-represented in the C-F subject pool including: *C. showae*, *C. rectus*, *C. curvus* and an uncharacterized phylotype. *C. gracilis* and *C. concisus* were over-represented in the C-A subject pool. *Cardiobacterium hominis, Haemophilus parainfluenzae* and *Lautropia mirabilis* were all over-represented in the C-F subject pool.

## Discussion

We developed a strategy (RAKE) to enrich 16S rDNA sequences through sequential removal of the most abundant nucleic acid species. We exploited the re-association kinetics of the dental plaque microbiome to sub-divide the re-associating DNAs into separate fractions prior to application of 16S rDNA sequence profiling. Compared to more traditional strategies that apply brute force sequencing to native metagenomic samples, the application of RAKE allowed a deeper and therefore more comprehensive characterization of the bacterial community. This method may also be applied to metagenomic DNAs where 10–100 fold normalization can be achieved (SNP, unpublished observation). The application of RAKE resulted in a near doubling of the OTUs identified.

### The Dental Plaque Biofilm Population Structure

Our survey of dental plaque biofilms supports the view that this ecological niche is a highly selective environment as we observe only four distinct phyla at an appreciable abundance. The Firmicutes are the dominant phyla. The presence of a relatively small number of genera (n = 36) representing a much larger number of species is thought to enhance the biochemical repertoire and complementarity of functions encoded within the dental plaque community. The sharing of “biochemical burden” amongst community members creates webs of mutualistic relationships. The functional redundancy inherent to related species may make it somewhat uncommon for any particular species to be “essential” to the community. This feature of the microbiota may explain the observed subject-to-subject variability observed in human microbiomes. The extent of the interpersonal variability in microbial composition of dental plaque within and across varied geography is largely uncharacterized but these factors are likely to directly contribute to the disparate results obtained by various association studies examining dental caries. The significance of the long tail structure in the population structure is unclear but may represent a reservoir of species that may modulate their abundance over time. It is conceivable that the maintenance of such reservoirs is evolutionarily favorable and a contributing component of the interpersonal variability of microbial communities.

### Genera of Dental Plaque Biofilms: Differential Abundance in C-F and C-A Subjects

The data summarized in **Figure 2** indicate that the majority (24 of 36) of genera present in dental plaque biofilms are over-represented in the C-F subject pool, whereas only 12 are over-represented in the C-A subject pool. Members of the genus *Streptococcus* are dominant in dental plaque biofilms, ten times more prevalent than the next most abundant genera. The modest over-representation of this genus in the C-A subject pool, is biochemically significant given the numerical dominance of this genera and its fermentative lifestyle and directly impinge on acid production. The next most highly represented genera included two Proteobacteria, *Campylobacter* and *Neisseria,* along with *Gemella* and the *Veillonella.* All but the *Veillonella* were over-represented in the C-F subject pool. Taken collectively, these five genera constitute ∼75% of the biofilm’s microbial biomass. The metabolic activities of species over-represented in the C-F subject pool are of potential interest as they are likely to play an important role in maintaining microbial composition and pH homeostasis**.** The biochemical basis underlying the caries phenotype is related to the balance of the metabolic pathways related to acid production, acid remediation via metabolism of strong acids (lactate) to weaker acids and finally, base production, generally in the form of microbially produced ammonium ions, carbonate or arginine [29], [37], [38]. The *Veillonella* are known to metabolize lactic acid to weaker acids. The fact that our survey indicates that *Veillonella* spp. are generally associated with heightened abundance in C-A subject pool suggests that they may serve as an “acid sink” in the ecosystem, allowing acidogenic species to continue to produce additional acid at a high rate through sugar metabolism and defines a significant example of caries-related mutualism. The remaining genera are mixed in their over-representation but the majority are over-represented in the C-F subject pool.

### Differential Species Abundance as Signatures of Dental Health and Disease

Despite the overall uniformity of phylotype representation in the C-F and C-A microbiota, we observed 87 phylotypes that were differentially represented in a statistically significant manner. Fourteen of the 87 differentially abundant phylotypes observed were found exclusively in either the C-F or C-A microbiome, however each instance corresponded to very low abundance phylotypes (<0.01%). We suggest that shifts in the community structure involving abundant species are more likely to be of biological significance with respect to caries. The significance to the observed diferences in abundance of low abundance species from a biochemical perspective is not clear. The phylotypes under-represented in C-A microbiomes may reflect their acid sensitivity and therefore represent useful diagnostic indicators of the pH trends in the dental plaque environment. The remaining 73 statistically significant differentially abundant phylotypes corresponded to a broad range of abundance. Slightly more than 50% of the differentially abundant phylotypes (52) were present at an abundance of 0.1% or higher.

The differentially represented Actinobacteria were predominantly (75%) over-represented in the C-A subject pool. These findings are consistent with a number of previously published findings [17]–[19]. *Corynebacterium matruchotii* is capable of both sugar utilization and the metabolism of organic acids like lactate and acetate [39]. The latter capability may explain their over-representation in the C-A microbiota. The relative balance of these activities in the context of dental biofilms has not been reported. This species has been previously described as a health-associated species, in contrast to our observations [23]. The differential representation of Bacteroidetes included a larger number of species. Ten of the twelve representative species were over-represented in the C-F subject pool. A group of three moderate-abundance (0.2–0.4%) Capnocytophaga spp. were over-represented in the C-F subject pool and may represent candidate signatures of dental health, consistent with a previous report associating *C. gingivalis* with dental health.

The high relative abundance of *Streptococcus* in our survey (52%) is somewhat higher than observed in other studies. One possible explanation for this disparity is that the dental biofilm communities were derived from subjects with limited fluoride exposure. We have observed similar results (48% *Streptococcus*) during an analysis of an adolescent cohort from Puerto Rico where fluoride was also absent in the drinking water (SNP, unpublished data). The differential representation of species belonging to the phylum Firmicutes were by far the most diverse, including 16 member genera. All four of the differentially represented Gemella spp. were over-represented in the C-F subject pool. *G. haemolysans* is an abundant species (2.7%) and *G. morbillorum* is moderately abundant (0.8%). The *Gemella* have not previously been associated with caries status. Both of the differentially represented *Abiotrophia* spp. were over-represented in the C-F subject pool, *A. defectiva* is an abundant species (2.5%). Both of the differentially represented *Granulicatella* spp. are abundant. *G. elegans* (2.6%) is over-represented in the C-A subject pool, and *G. adjacens* (0.9%) is over-represented in the C-F subject pool. In total, 18 *Streptococcus* spp. were differentially abundant and 13 of those were over-represented in the C-A subject pool. More significant than the number is the fact that together these 13 species comprise nearly 50% of the dental plaque biomass. The distinction between dental health and disease may have less to do with the overall abundance of *Streptococcus* spp. but rather the relative balance between strongly acidogenic species and less acidogenic species and their relative rates (velocity) of acid production. It is well established that *S. mutans* and *S. sobrinus* are among the most acidogenic species present in dental plaque. Likewise *S. mitis* isolates, while variable in their acid production, include highly acidogenic strains [40]. It is unclear why *S. sanguinis* and *S. mitis,* species normally associated with dental health, are over-represented in the C-A subject pool. Similarly, *S. cristatus* and *S. gordonii* have previously been associated with dental health [23], whereas we observe associations of these species with caries activity. These conflicting results may reflect strain level phenotypic variation that serves to elevate the acidogenicity and/or aciduricity of the represented species. The validity of this speculation however will require experimentation. The strong over-representation of *S. mutans* in C-A subjects is consistent with numerous reports but also illustrates that despite the sampling of dental plaque from all tooth surfaces, the *S. mutans* signature remains apparent. The *Veillonella* are asaccharolytic and metabolizes the products generated by primary fermentation for energy. Their ability to metabolize lactate to weaker acids suggests that its metabolic activities may be protective and associated with caries health. Our results suggest that *V. parvula,* an abundant species through its lactate metabolism may serve as an acid sink in dental biofilms and further enable sugar metabolism of acidogenic and aciduric species. These findings are consistent with those recently reported by Gross and colleagues and with *in vitro* cultivation experiments demonstrating that *S. mutans* produced more acid when co-cultivated with *V. parvula* compared to when *S. mutans* was grown as a mono-culture [41].

Among the Fusobacteria, all three differentially abundant phylotypes were over-represented in the C-F subject pool. Among the differentially abundant Proteobacteria were six *Neisseria* spp. Two-thirds of these were over-represented in the C-F subject pool, although the most abundant species, *N. subflava* was over-represented in the C-A subject pool. Finally six *Campylobacter* spp. were differentially represented. Among the three most prevalent, *C. showae* is over-represented in the C-F subject pool, whereas both *C. gracilis* and *C. concisus* were over-represented in the C-A subject pool. It is known that individual species within both the *Neisseria* and *Campylobacter* are capable of metabolizing sugar to lactate and many members of these genera are also capable of metabolizing lactate to lower pKa acids [42]. As additional reference genome sequences pertaining to the oral cavity continue to accumulate [43], we will be able to relate metabolic coding potential of genomes to the dental caries phenotype.

The association of *S. mutans* and lactobacilli in the development and progression of dental caries is strong, however there are several documented cases of caries in human subjects that are independent of *S. mutans* and Lactobacilli [5]–[7], [9], [10], [18]. Recently, Kanasi et al., reported that *Bifidobacterium* spp. were associated with severe caries in children [22]. We failed to identify any significant Lactobacilli or *Bifidobacterium* spp. in our survey. When we compared the sequences of available 16S rDNAs corresponding to these phyla we conclude that the primer pairs used here to amplify 16S rDNA should have resulted in amplification of Lactobaccili but may not have efficiently amplified *Bifidobacterium* spp. due to several mismatches noted in the priming sites.

Despite the growing number of published studies examining the species profiles associated with caries status there appears to be limited agreement in many of the results obtained. Furthermore, the methods used and depth of characterization achieved in each study make effective data comparisons difficult to perform. The recent report by Gross et al., represents a significant study illustrating the interpersonal variability of dental biofilm microbiota [23]. We hypothesize that the human dental plaque microbiota and others contain a large number of member species/strains that are functionally inter-changeable components within their respective community. Our study emphasizes that it is the shift in abundance of groups of species rather than the “appearance” of novel cariogenic species or the pathogenicity of a single species that best describes the distinction between C-F and C-A microbiota. The large number of potentially “dispensable” species present in dental plaque biofilm may strongly confound association studies. Future efforts to enable the recognition of “synonymous species” in microbiomes may be critical for establishing an understanding of those that provide protective qualities to maintain dental health compared to those that can contribute to disease.

It should be emphasized that our preliminary profiling of C-A and C-F subjects, reflect a census of two microbiomes comprised of a small number of human subjects. In order to establish statistically rigorous species associations as diagnostic indicators of dental health or disease, the profiling of larger numbers of samples derived from individuals rather than pools will be required. The use of twins discordant for dental caries together with longitudinal studies may help to elucidate these intriguing concepts. In addition, we anticipate that metagenomic analysis of species and their associated gene and metabolite expression associated with dental health and disease will further hasten our ability to reduce oral health disparities in the general population.

## Supporting Information

Figure S1 **Population Structure of Dental Plaque Biofilm Microbiome.** The dental plaque microbiome is comprised of a small group of 16 highly abundant OTUs (>1% abundance, blue) followed by 117 OTUs representing moderately abundant phylotypes (>0.1% abundance, green), 500 OTUs representing low abundance phylotypes (>0.01% yellow) and finally a large and incompletely characterized group of very low abundance OTUs (>0.001%, red) constituting a long tail in the population structure.
(TIF)

Table S1 **Phylotypes Differentially Represented in C-F and C-A Dental Biofilms (continued).** Differentially abundant phylotypes in C-F and C-A microbiota.
(DOC)
