# Supplementary figures and images for: The Dental Plaque Microbiome in Health and Disease
Source: PLoS One. 2013 Mar 8;8(3):e58487. doi: 10.1371/journal.pone.0058487 (PMC3592792; doi:10.1371/journal.pone.0058487)

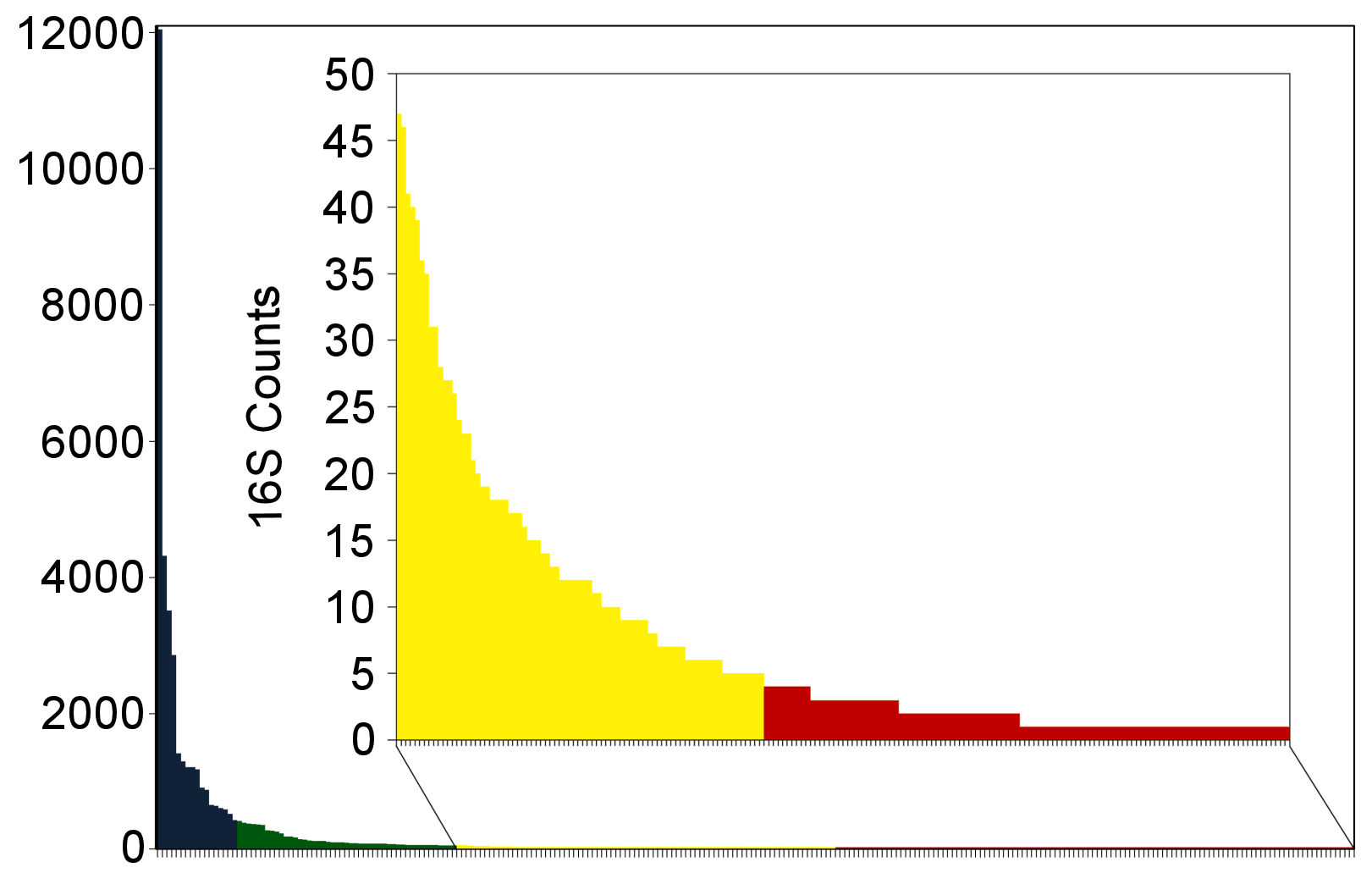

Supplement: Figure S1 — Population Structure of Dental Plaque Biofilm Microbiome. The dental plaque microbiome is comprised of a small group of 16 highly abundant OTUs (>1% abundance, blue) followed by 117 OTUs representing moderately abundant phylotypes (>0.1% abundance, green), 500 OTUs representing low abundance phylotypes (>0.01% yellow) and finally a large and incompletely characterized group of very low abundance OTUs (>0.001%, red) constituting a long tail in the population structure. (TIF) [file pone.0058487.s001.tif]
