# Supplementary material for: The Dental Plaque Microbiome in Health and Disease
Source: PLoS One. 2013 Mar 8;8(3):e58487. doi: 10.1371/journal.pone.0058487 (PMC3592792; doi:10.1371/journal.pone.0058487)
Supplement: Table S1 — Phylotypes Differentially Represented in C-F and C-A Dental Biofilms (continued). Differentially abundant phylotypes in C-F and C-A microbiota. (DOC) [file pone.0058487.s002.doc]

**Table 1S. Phylotypes Differentially Represented in C-F and C-A Dental Biofilms.**

|  | | **Total** | **C-F** | **C-A** | **Phylotype** | **p-value** | **FDR** | | --- | --- | --- | --- | --- | --- | | **47198** | **21705** | **25493** | Bacteria |  |  | | 4325 | 1288 | 3037 | *Streptococcus sanguinis* | 0 | 0 | | 3520 | 1205 | 2315 | *Veillonella parvula* | 0 | 0 | | 1220 | 697 | 523 | *Granulicatella elegans* | 0 | 0 | | 1220 | 381 | 839 | *Streptococcus gordonii* | 0 | 0 | | 1189 | 867 | 322 | *Abiotrophia defectiva* | 0 | 0 | | 865 | 168 | 697 | *Campylobacter gracilis* | 0 | 0 | | 643 | 544 | 99 | *Campylobacter showae* | 0 | 0 | | 633 | 420 | 213 | *Streptococcus infantis* | 0 | 0 | | 601 | 146 | 455 | *Streptococcus constellatus* | 0 | 0 | | 578 | 8 | 570 | *Streptococcus mutans* | 0 | 0 | | 359 | 269 | 90 | *Fusobacterium nucleatum* | 0 | 0 | | 350 | 266 | 84 | *Selenomonas infelix* | 0 | 0 | | 250 | 190 | 60 | *Streptococcus australis* | 0 | 0 | | 141 | 118 | 23 | *Catonella morbi* | 0 | 0 | | 93 | 85 | 8 | *Veillonella criceti* | 0 | 0 | | 81 | 73 | 8 | *Capnocytophaga leadbetteri* | 0 | 0 | | 511 | 158 | 353 | *Campylobacter concisus* | 6.358E-12 | 2.500E-11 | | 225 | 54 | 171 | *Kingella denitrificans* | 3.282E-11 | 1.220E-10 | | 76 | 62 | 14 | *Veillonella sp.* | 4.642E-10 | 1.640E-09 | | 48 | 1 | 47 | *Streptococcus sobrinus* | 1.020E-09 | 3.430E-09 | | 62 | 52 | 10 | *Porphyromonas sp.* | 2.109E-09 | 6.780E-09 | | 88 | 68 | 20 | *Capnocytophaga sputigena* | 3.765E-09 | 1.160E-08 | | 1302 | 702 | 600 | *Gemella haemolysans* | 5.810E-09 | 1.710E-08 | | 96 | 72 | 24 | *Neisseria elongata* | 1.133E-08 | 3.200E-08 | | 48 | 41 | 7 | *Abiotrophia sp.* | 4.158E-08 | 1.130E-07 | | 77 | 59 | 18 | *Parvimonas micra* | 6.722E-08 | 1.760E-07 | | 1421 | 557 | 864 | *Neisseria subflava* | 1.845E-07 | 4.660E-07 | | 26 | 25 | 1 | *Prevotella sp.* | 2.837E-07 | 6.920E-07 | | 101 | 72 | 29 | *Lautropia mirabilis* | 3.270E-07 | 7.710E-07 | | 49 | 40 | 9 | *Fusobacterium canifelinum* | 5.468E-07 | 1.250E-06 | | 74 | 55 | 19 | *Neisseria sp.* | 9.834E-07 | 2.170E-06 | | 370 | 124 | 246 | *Streptococcus salivarius* | 1.344E-06 | 2.880E-06 | | 47 | 38 | 9 | *Selenomonas dianae* | 1.601E-06 | 3.330E-06 | | 72 | 53 | 19 | *Gemella sanguinis* | 2.517E-06 | 5.090E-06 | | 273 | 88 | 185 | *Streptococcus intermedius* | 4.817E-06 | 9.410E-06 | | 58 | 44 | 14 | *Neisseria bacilliformis* | 4.925E-06 | 9.410E-06 | | 12047 | 5747 | 6300 | *Streptococcus mitis* | 1.168E-05 | 2.170E-05 | | 76 | 16 | 60 | *Leptotrichia wadei* | 1.271E-05 | 2.250E-05 | | 31 | 26 | 5 | *Campylobacter rectus* | 2.300E-05 | 3.970E-05 | | 2864 | 1424 | 1440 | *Streptococcus oralis* | 3.526E-05 | 5.940E-05 | | 112 | 73 | 39 | *Porphyromonas catoniae* | 4.503E-05 | 7.400E-05 | | 18 | 0 | 18 | *Corynebacterium matruchotii* | 9.021E-05 | 1.450E-04 | | 71 | 49 | 22 | *Leptotrichia sp.* | 9.780E-05 | 1.540E-04 | | 19 | 17 | 2 | *Prevotella tannerae* | 0.0001423 | 2.160E-04 | | 46 | 34 | 12 | *Eubacterium brachy* | 0.0001434 | 2.160E-04 | | 27 | 3 | 24 | *Megasphaera micronuciformis* | 0.0002756 | 4.060E-04 | | 179 | 106 | 73 | *Capnocytophaga granulosa* | 0.0003729 | 0.0005382 | | 20 | 17 | 3 | *Leptotrichia shahii* | 0.0004627 | 0.0006544 | | 28 | 22 | 6 | *Firmicutes oral* | 0.0005390 | 0.0007473 | | 361 | 197 | 164 | *Gemella morbillorum* | 0.0010203 | 0.0013873 | | 23 | 3 | 20 | *Selenomonas genomosp.* | 0.0015200 | 0.0020279 | | 8 | 8 | 0 | *Campylobacter curvus* | 0.0021725 | 0.0028447 | | 14 | 12 | 2 | *Streptococcus oligofermentans* | 0.0028546 | 0.0036700 | | 263 | 97 | 166 | *Streptococcus parasanguinis* | 0.0029682 | 0.0037479 | | 64 | 41 | 23 | *Haemophilus parainfluenzae* | 0.0036915 | 0.0045793 | | 7 | 7 | 0 | *Propionibacterium propionicum* | 0.0041366 | 0.0050430 | | 36 | 8 | 28 | *Actinomyces naeslundii* | 0.0042083 | 0.0050435 | | 85 | 26 | 59 | *Eubacterium saburreum* | 0.0043558 | 0.0051333 | | 21 | 16 | 5 | *Neisseria* spp. | 0.0054735 | 0.0063447 | | 9 | 0 | 9 | *Mitsuokella sp.* | 0.0056329 | 0.0064242 | | 6 | 6 | 0 | *Mogibacterium timidum* | 0.0079353 | 0.0086323 | | 6 | 6 | 0 | *Prevotella intermedia* | 0.0079353 | 0.0086323 | | 6 | 6 | 0 | *Simonsiella muelleri* | 0.0079353 | 0.0086323 | | 68 | 42 | 26 | *Eubacterium sp.* | 0.0089906 | 0.0096322 | | 12 | 10 | 2 | *Cardiobacterium hominis* | 0.0094282 | 0.0099502 | | 9 | 8 | 1 | *Prevotella nanceiensis* | 0.0098035 | 0.0101942 | | 41 | 27 | 14 | *Peptostreptococcus stomatis* | 0.0106646 | 0.0109288 | | 11 | 1 | 10 | *Prevotella oulorum* | 0.0140640 | 0.0141112 | | 901 | 378 | 523 | *Streptococcus cristatus* | 0.0141691 | 0.0141112 | | 24 | 17 | 7 | *Clostridiales bacterium* | 0.0145689 | 0.0141688 | | 7 | 0 | 7 | *Prevotella salivae* | 0.0146278 | 0.0141688 | | 5 | 5 | 0 | *Dialister pneumosintes* | 0.0153728 | 0.0143027 | | 5 | 5 | 0 | *Fusobacterium periodonticum* | 0.0153728 | 0.0143027 | | 5 | 5 | 0 | *Prevotella saccharolytica* | 0.0153728 | 0.0143027 | | 6 | 0 | 6 | *Streptococcus equi* | 0.0238005 | 0.0218562 | | 4 | 4 | 0 | *Gemella bergeri* | 0.0301892 | 0.0270211 | | 4 | 4 | 0 | *Neisseria lactamica* | 0.0301892 | 0.0270211 | | 15 | 11 | 4 | *Solobacterium moorei* | 0.0335514 | 0.0296551 | | 7 | 6 | 1 | *Campylobacter sp.* | 0.0349339 | 0.0301239 | | 7 | 6 | 1 | *Eubacterium yurii* | 0.0349339 | 0.0301239 | | 411 | 168 | 243 | *Granulicatella adiacens* | 0.0367778 | 0.0313319 | | 56 | 18 | 38 | *Veillonella atypica* | 0.0375293 | 0.0315914 | | 5 | 0 | 5 | *Streptococcus dentirousetti* | 0.0390773 | 0.0325076 | | 12 | 2 | 10 | *Actinomyces sp.* | 0.0415286 | 0.0337525 | | 12 | 2 | 10 | *Streptococcus peroris* | 0.0415286 | 0.0337525 | | 12 | 9 | 3 | *Centipeda periodontii* | 0.0437129 | 0.0351242 | | 52 | 31 | 21 | *Streptococcus suis* | 0.0485019 | 0.0385343 | |  |  |  |  |
| --- | --- | --- | --- | --- | --- | --- | --- | --- | --- | --- | --- | --- | --- | --- | --- | --- | --- | --- | --- | --- | --- | --- | --- | --- | --- | --- | --- | --- | --- | --- | --- | --- | --- | --- | --- | --- | --- | --- | --- | --- | --- | --- | --- | --- | --- | --- | --- | --- | --- | --- | --- | --- | --- | --- | --- | --- | --- | --- | --- | --- | --- | --- | --- | --- | --- | --- | --- | --- | --- | --- | --- | --- | --- | --- | --- | --- | --- | --- | --- | --- | --- | --- | --- | --- | --- | --- | --- | --- | --- | --- | --- | --- | --- | --- | --- | --- | --- | --- | --- | --- | --- | --- | --- | --- | --- | --- | --- | --- | --- | --- | --- | --- | --- | --- | --- | --- | --- | --- | --- | --- | --- | --- | --- | --- | --- | --- | --- | --- | --- | --- | --- | --- | --- | --- | --- | --- | --- | --- | --- | --- | --- | --- | --- | --- | --- | --- | --- | --- | --- | --- | --- | --- | --- | --- | --- | --- | --- | --- | --- | --- | --- | --- | --- | --- | --- | --- | --- | --- | --- | --- | --- | --- | --- | --- | --- | --- | --- | --- | --- | --- | --- | --- | --- | --- | --- | --- | --- | --- | --- | --- | --- | --- | --- | --- | --- | --- | --- | --- | --- | --- | --- | --- | --- | --- | --- | --- | --- | --- | --- | --- | --- | --- | --- | --- | --- | --- | --- | --- | --- | --- | --- | --- | --- | --- | --- | --- | --- | --- | --- | --- | --- | --- | --- | --- | --- | --- | --- | --- | --- | --- | --- | --- | --- | --- | --- | --- | --- | --- | --- | --- | --- | --- | --- | --- | --- | --- | --- | --- | --- | --- | --- | --- | --- | --- | --- | --- | --- | --- | --- | --- | --- | --- | --- | --- | --- | --- | --- | --- | --- | --- | --- | --- | --- | --- | --- | --- | --- | --- | --- | --- | --- | --- | --- | --- | --- | --- | --- | --- | --- | --- | --- | --- | --- | --- | --- | --- | --- | --- | --- | --- | --- | --- | --- | --- | --- | --- | --- | --- | --- | --- | --- | --- | --- | --- | --- | --- | --- | --- | --- | --- | --- | --- | --- | --- | --- | --- | --- | --- | --- | --- | --- | --- | --- | --- | --- | --- | --- | --- | --- | --- | --- | --- | --- | --- | --- | --- | --- | --- | --- | --- | --- | --- | --- | --- | --- | --- | --- | --- | --- | --- | --- | --- | --- | --- | --- | --- | --- | --- | --- | --- | --- | --- | --- | --- | --- | --- | --- | --- | --- | --- | --- | --- | --- | --- | --- | --- | --- | --- | --- | --- | --- | --- | --- | --- | --- | --- | --- | --- | --- | --- | --- | --- | --- | --- | --- | --- | --- | --- | --- | --- | --- | --- | --- | --- | --- | --- | --- | --- | --- | --- | --- | --- | --- | --- | --- | --- | --- | --- | --- | --- | --- | --- | --- | --- | --- | --- | --- | --- | --- | --- | --- | --- | --- | --- | --- | --- | --- | --- | --- | --- | --- | --- | --- | --- | --- | --- | --- | --- | --- | --- | --- | --- | --- | --- | --- | --- | --- | --- | --- | --- | --- | --- | --- | --- | --- | --- | --- | --- | --- | --- | --- | --- | --- | --- | --- | --- | --- | --- | --- | --- | --- | --- | --- | --- | --- | --- | --- | --- | --- | --- | --- | --- | --- | --- | --- | --- | --- | --- | --- | --- | --- | --- | --- | --- | --- | --- | --- | --- | --- | --- | --- | --- | --- | --- | --- | --- | --- | --- | --- |
